# Validation of a cloud-based tele-stroke system reliability in determining national institutes of health stroke scale scores for acute ischemic stroke screening in the emergency department

**DOI:** 10.3389/fneur.2022.973165

**Published:** 2022-09-20

**Authors:** Mor Saban, Anner Moskovitz, Sona Ohanyan, Anna Reznik, Marc Ribo, Rotem Sivan-Hoffmann

**Affiliations:** ^1^The Gertner Institute for Health Policy and Epidemiology, Ramat-Gan, Israel; ^2^The Ruth and Bruce Rappaport Faculty of Medicine, Technion Israel Institute of Technology, Haifa, Israel; ^3^Department of Neurology, Rambam Healthcare Campus, Haifa, Israel; ^4^Department of Interventional Neuroradiology, Vall d'Hebron University Hospital, Barcelona, Spain; ^5^Department of Interventional Neuroradiology, Rambam Healthcare Campus, Haifa, Israel

**Keywords:** stroke, National Institute of Health Stroke Scale, large vessel occlusion, tele-stroke, remote diagnosis

## Abstract

**Background and purpose:**

The National Institutes of Health Stroke Scale (NIHSS) is the most recommended tool for objectively quantifying the impairment caused by a suspected stroke. Nevertheless, it is mainly used by trained neurologists in the emergency department (ED). To bring forward the NIHSS to the pre-hospital setting, a smartphone-based Telestroke system was developed. It captures the full NIHSS by video, transmits it off-line, and enables assessment by a distant stroke physician. We aimed to compare the reliability of an NIHSS score determined by a neurologist from afar, using the platform with a standard NIHSS assessment performed in the emergency departments.

**Methods:**

A multi-center prospective study was conducted in two centers (Vall d'Hebron, Barcelona, and Rambam, Israel). Patients admitted to the ED with suspected stroke had a neurological exam based on the NIHSS, while being recorded by the system. A skilled neurologist rated the NIHSS according to the videos offline. The results were compared with the NIHSS score given by a neurologist at the bedside.

**Results:**

A total of 95 patients with suspected stroke were included. The overall intraclass correlation coefficient was 0.936 (0.99 in VdH and 0.84 in Rambam), indicating excellent and good reliability, respectively.

**Conclusion:**

Remote stroke assessment based on the NIHSS, using videos segments collected by a dedicated platform, installed on a standard smartphone, is a reliable measurement as compared with the bedside evaluation.

## Introduction

Management of acute ischemic stroke (AIS) has changed dramatically over the last few years, following evidence on the superiority of endovascular treatment (EVT) in the treatment of large vessel occlusion (LVO) in patients treated within 6–8 hours from onset of symptoms (1). The window for treatment was further extended to 24 hours following trials that demonstrated the efficacy of EVT for selected patients in time frames up to 24 hours (2, 3). The immediate consequence was an increase in the number of patients eligible for EVT, requiring secondary transfer of those patients from primary stroke centers to comprehensive stroke centers, capable of preforming EVT.

Endovascular treatment requires accurate and rapid diagnosis in the pre-hospital setting, as it is only indicated in specific patients with LVO, making up a small percentage of AIS cases (4), and its beneficial effects are highly time dependent. The main ways of care for patients with suspected AIS are either the “drip-and-ship” approach or the “mothership” approach. The former consists of evacuating patients to the nearest primary stroke center to perform imaging, start intravenous thrombolysis (IVT), when indicated, and transfer patients for EVT. The mothership approach consists of evacuating patients directly to a comprehensive stroke center (5).

Since the mothership approach reduces the transfer time of patients to EVT, it was found to be beneficial for patients suffering from an LVO (6, 7). On the contrary, in non-LVO stroke and stroke mimics cases, which could be treated in the primary stroke centers, it may be redundant or even delay initiation of medical treatment without any added benefit.

Of the existing diagnostic tools that accurately distinguish LVO cases from non-LVO ones, the National Institutes of Health Stroke Scale (NIHSS) is the most recommended tool by healthcare providers to objectively quantify the neurological impairment caused by a suspected stroke (8). Until recently it was assumed to be too complicated and time-consuming to be used by paramedics in the field (9). Several shorter derivatives were developed to identify patients with a high probability of LVO in the pre-hospital setting. However, there is no clear evidence for superiority of one scale over the others. So far, no scale was found to determine the presence vs. absence of LVO with both high sensitivity and specificity (10).

To resolve this issue, a smartphone-cloud-based Artificial Intelligence (AI) supported Telestroke system was developed. The aim of this system is to evaluate the patient's neurological status, assessment of stroke severity, and prediction of LVO as the cause of stroke. The system first captures the full NIHSS by video while presenting instructions for the neurological exam on the screen to the medical professional operating it on a standard smartphone, and storing the data on a secure cloud storage. The system then enables a distant stroke physician to assess the patient's status manually, without the need of online presence or real time interface with the patient. It is the first step toward an AI decision support tool.

Recent articles exploring the use of a smartphone application for in-hospital stroke treatment have shown promising results with reductions of patient management times, satisfaction of medical staff and similar neurological outcomes (11, 12). Those results strengthen the hypothesis that an application built for pre-hospital and ED decision making could aid medical personnel in such positions.

In this study, we aim to evaluate the reliability of an NIHSS score by a remote neurologist rating captured video segments examinations, compared with a standard NIHSS assessment done by a bedside neurologist in the emergency department.

## Methods

### Study design and participants

**Figure 1:**
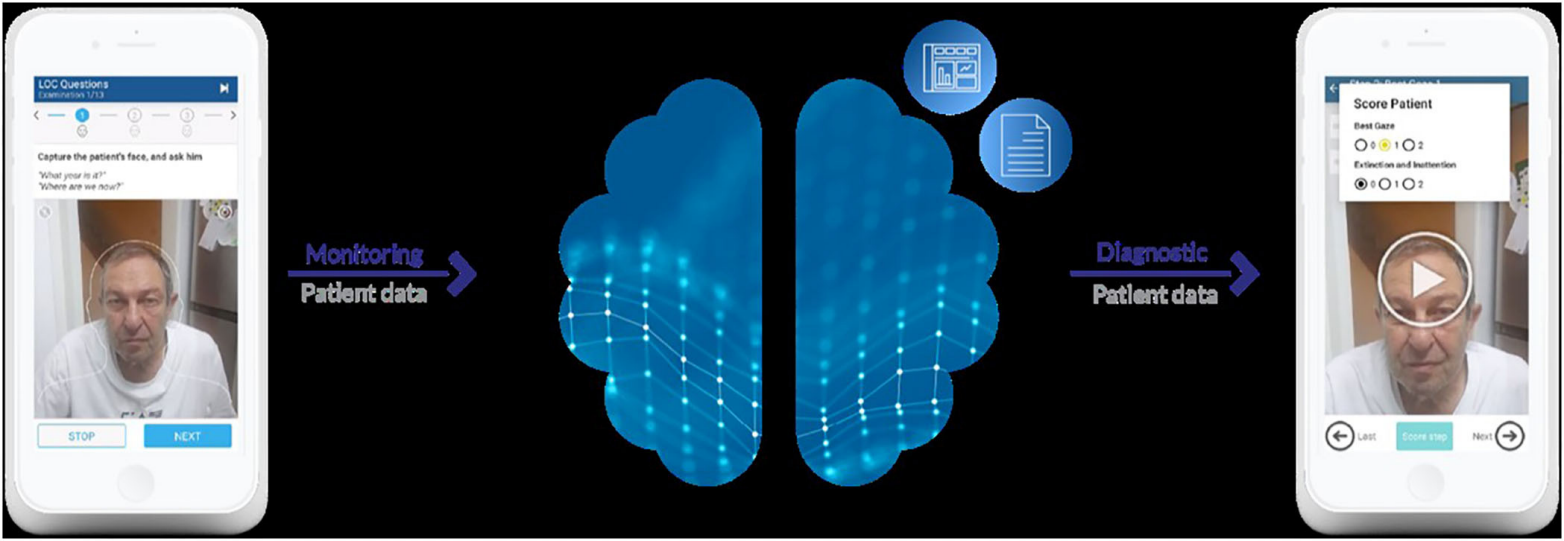
The left part shows a patient being instructed through the neurological exam with instructions for the researcher/medical professional shown on the screen. The right part shows the app used be the neurologist rating the patient's state based on the video segments.

This multi-center prospectively controlled clinical trial includes patients admitted to the EDs in the Rambam Hospital in Haifa, Israel and the Vall d'Hebron Hospital in Barcelona, Spain. Inclusion criteria were: (1) age over 18 years, (2) symptoms suggestive of AIS, (3) had not received treatment (IVT or EVT) prior to the examination. Exclusion criteria were: (1) patient intubated upon arrival, (2) time from stroke symptoms onset >24 hours, (3) post-treatment (IVT or EVT), (4) patient diagnosed in the ED as suffering from a different condition which could simulate signs of AIS (such as hypoglycemia). Following the introduction by the researcher and signing an informed consent form, each subject was allocated a number by the application for anonymization. Next, the subject was instructed by the researcher through a neurological exam based on the NIHSS with simple instructions presented on the screen, while being recorded. The application uses the smartphone's built-in camera and microphone, to capture the clinical signs of the patient, and the data collected is uploaded in segments of the exam, using a secured network to the external server (Figure 1). The video is then sent in short segments representing each of the NIHSS tests to a remote physician, using an application enabling the stroke team to watch the full exams from a far. An independent neurologist blinded to the score obtained on site rated the NIHSS according to the videos remotely and offline. Both examinations were performed in a similar time frame before treatment (thrombolysis or EVT). The results were compared with the NIHSS score assigned by one of five independent bedside neurologists from the relevant hospital as part of the routine clinical practice. All the raters are NIHSS-certified.

### Ethical considerations

The study protocol was approved by the Institutional Human Subjects Ethics Committee (IRB-0187-18). All the data were encrypted and transmitted in a highly secure manner, adhering to the Health Insurance Portability and Accountability Act (HIPAA) regulations.

### Statistical methods

In order to validate the reliability of the collected data, the digitalized total score of the NIHSS were correlated with the results of the standard, bedside NIHSS examination.

Inter-rater reliability of the total NIHSS score was quantified using an intraclass correlation coefficient (ICC) model (2,1) (13), namely, two-way random effects, absolute agreement, and single rates/measurement.

Inter-rater reliability of individual item scores was quantified using a conservative weighted Kappa (wK) coefficient. Based on the 95% CI of the ICC estimate, ICC values <0.5 are indicative of poor reliability, values between 0.5 and 0.75 indicate moderate reliability, values between 0.75 and 0.9 indicate good reliability, and values >0.90 indicate excellent reliability (10). We also examined if the difference between scores set from afar and from bedside was over two NIHSS points by using one sample Student's *t*-test. Following that, we calculated the ICC considering successful correlation if bedside rating was within 2 points of the remote rating.

Sensitivity to detect change of the total NIHSS score was estimated at different levels of confidence using the Minimal Detectable Difference (MDD) and the Bland–Altman plots. Factors associated with absolute disagreement on individual scale items and magnitude of disagreement on the total NIHSS score between raters were investigated using logistic and linear regression, respectively (14).

The level of significance for all the statistical analysis was 5%. The data analysis was performed using the Statistical Package for Health & Welfare Science for Windows (SPSS, version 25.0, Chicago, IL, USA).

## Results

A total of 95 patients with suspected AIS were included, 44 in Rambam and 51 from Vall d'Hebron. Patient characteristics and results are summarized in Table 1. Total number of patients subsequently diagnosed with LVO was 32 (33.6%) with no differences in prevalence between centers. The percentage of males was slightly higher in Rambam compared with Vall d'Hebron. ICC (2,1) overall and in Vall d'Hebron was higher than 0.9, and for Rambam higher than 0.75, indicating excellent and good reliability, respectively (10).

**Table 1:** Patient characteristics and intraclass correlation coefficient (ICC) of total National Institute of Health Stroke Scale score.

**Study site**	**Total**	**Rambam**	**Vall d'Hebron**
N	95	44	51
Age	69.93 ± 14.11	69.89 ± 13.77	69.06 ± 15.72
Male	50 (52.6%)	27 (61.4%)	23 (45.1%)
NIHSS	5 [IQR, 5]	5 [IQR, 6]	4 [IQR, 7]
LVO	32 (33.7%)	15 (34.1%)	17 (33.3%)
ICC (2,1)	0.936^*^	0.847^*^	0.991^*^

Age is presented as mean ± standard deviation. Male and LVO rates are presented as absolute number and percentage. NIHSS is presented as median. IQR, Interquartile range; LVO, Large vessel occlusion; N, Number of patients; NIHSS, National Institute of Health Stroke Scale.

* *P*-value < 0.001.

**Figure 2:**
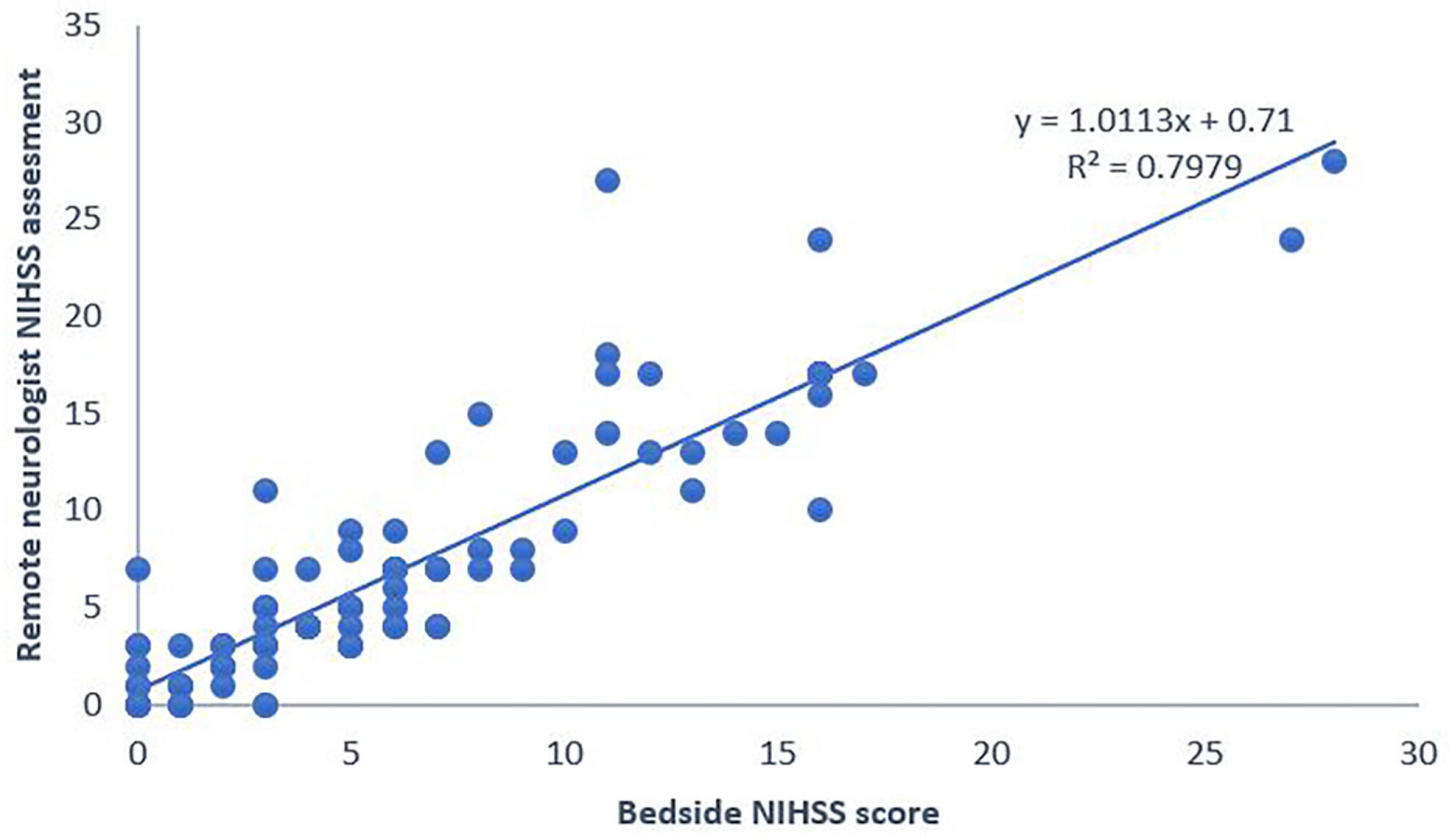
National Institute of Health Stroke Scale (NIHSS) scores set using the system remotely compared with those set at bedside. NIHSS, National Institute of Health Stroke Scale.

One sample Student's *t*-test showed the total NIHSS scores set from afar compared with those set at bedside did not differ significantly more than two points (*p* = 0.325, Figure 2). We then calculated the ICC (2,1) adjusted for the NIHSS scores with disagreement within two NIHSS points. The results indicate excellent reliability (ICC = 0.939).

Subscale analysis for the individual reliability (wK) is summarized in Table 2. wK scores ranged from 0.285 to 0.646, indicating fair to the substantial agreement, with the highest individual reliability observed in item 1b Level of Consciousness (LOC) Questions, followed by item 3, Visual and item 11, Extinction and Inattention. The lowest was in item 7, Limb Ataxia. *P*-value for all the Kappa scores was <0.001.

**Table 2:** Interrater agreement (weighted Kappa) on the subscales of the NIHSS.

**Subscale**	**wK (95% CI; *n* = 95)**
1a Level of consciousness	0.326^*^ (0.045–0.607)
1b LOC questions	0.646^*^ (0.511–0.782)
1c LOC commands	0.407^*^ (0.162–0.652)
2 Gaze	0.567^*^ (0.37–0.764)
3 Visual	0.633^*^ (0.477–0.79)
4 Facial Palsy	0.461^*^ (0.325–0.597)
5a Motor arm: Left arm	0.503^*^ (0.355–0.65)
5b Motor arm: Right arm	0.355^*^ (0.173–0.538)
6a Motor leg: Left leg	0.432^*^ (0.278–0.586)
6b Motor leg: Right leg	0.398^*^ (0.201–0.596)
7 Limb ataxia	0.285^*^ (0–0.57)
8 Sensory	0.491^*^ (0.306–0.676)
9 Language aphasia	0.499^*^ (0.339–0.658)
10 Dysarthria	0.458^*^ (0.314–0.602)
11 Extinction and inattention	0.626^*^ (0.432–0.819)

CI, Confidence interval; LOC, Level of consciousness; wK, weighted Kappa.

* *p*-Value < 0.001.

One sample Student's *t*-test showed the difference between scores differs from 0 significantly (*p* < 0.05), therefore, MDD and Bland–Altman plot were unnecessary.

Intra-rater reliability for the independent remote neurologists who rated the NIHSS according to the offline videos and for the five bedside neurologists who rated the NIHSS as part of the routine clinical practice, was high for both the raters' groups (Intraclass Correlation Coefficient: 0.89 and 0.87, respectively).

## Discussion

In this study, we compared the reliability of an NIHSS score determined by a neurologist from afar, using smartphone cloud-based platform, to a standard NIHSS assessment done by a bedside neurologist in the emergency department and found it to be high, with the most scores falling within two points of those set at bedside.

Our results suggest that the total NIHSS determined by a neurologist from afar, using recorded, cloud-based video-segments of the exam, has a good to excellent reliability compared with a standard NIHSS assessment performed at bedside.

Interrater agreement on the total NIHSS score was consistent with preceding studies on real patients (0.91–0.98) (15–17). Similar variability in the subscale items' reliabilities was seen, with lowest wK ranging between −0.07 and 0.35 in most articles, and the highest wK ranging between 0.83 and 1.

Agreement between raters ranged from fair to substantial, with most wK values falling in the moderate agreement range.

The dissimilarities probably stem from the target study population characteristics and scale, exclusion criteria, and technological variations. Shafqat et al. (18) showed comparable results for all the subscales except motor arm (5 a and b) and motor leg (6 a and b), which may be attributed to their use of a dual camera system (wide angle and zoom) in contrast to our use of smartphone camera.

The lowest reliability was observed in limb ataxia (7). Low reliability of limb ataxia was noted in many studies using telemedicine on patients with stroke (wK −0.07–0.35 in most studies), as well as in older studies conducted by Goldstein et al. (19) and Brott et al. (20) who studied the interrater agreement on bedside administered NIHSS. This finding suggests that this NIHSS item may has intrinsic inferior interrater agreement.

In most previous studies, the remote neurologist directed the examination while determining the NIHSS score in real time (21–24). The use of recordings rather than real time examination is an important part of the validation process of the platform, aiming for future AI predictions of stroke and LVO probability base on it.

The differences between the two centers need to be noted. The NIHSS at bedside was done in Rambam by on-call neurologists, usually residents. In Vall d'Hebron, it was performed by skilled neurologists from the stroke team. The NIHSS assessment from afar was done in both centers by skilled senior neurologists. Superior results in Vall d'Hebron suggest that the evaluation carried out through the system by a senior neurologist might be more accurate than the one done at bedside by a resident. If this is true, using the system, will increase the accuracy of stroke detection.

One limitation of this study is the lack of stroke mimics that might have decreased the variance and thus improved the results. Further research is needed for validation of the use of telestroke systems in such conditions, as well as their effect on treatment and outcomes for patients with diverse illnesses. Another limitation comes from the point that this study was built for validation of the reliability of the system, and therefore, time intervals were not assessed. Future research, based on the findings presented in this article, could include comparison of standard protocol vs. the usage of smartphone-based telestroke systems in measures such as time elapsed in determination of stroke probability and comparison of patients' outcomes.

This study indicates that the NIHSS determination from afar on captured video segments is as good as (and maybe even superior to) bedside assessment. Thus, enabling the possibility for future studies regarding assessment of stroke and LVO utilizing artificial intelligence assisted program and a standard smartphone, without the need for special equipment or hardware.

## Conclusion

Remote stroke assessment based on the NIHSS, using videos collected by a dedicated platform, installed on a standard smartphone, is a reliable measurement as compared with bedside evaluation.

## Data availability statement

The original contributions presented in the study are included in the article/supplementary material, further inquiries can be directed to the corresponding author.

## Ethics statement

The studies involving human participants were reviewed and approved by the Institutional Human Subjects Ethics Committee (IRB-0187-18). The patients/participants provided their written informed consent to participate in this study. Written informed consent was obtained from the individual(s) for the publication of any potentially identifiable images or data included in this article.

## Author contributions

MS, AM, AR, and RS-H conceptualized and designed the study, drafted the initial manuscript, and reviewed and revised the manuscript. MS, AM, SO, MR, and RS-H designed the methods section, analyzed the data, and reviewed and revised the manuscript. MS, MR, and RS-H critically reviewed the manuscript for important intellectual content. All authors interpreted the data and edited and approved the final article.

## Funding

The authors declare that this study received funding from CVAID Company. The funder was not involved in the study design, collection, analysis, interpretation of data, the writing of this article, or the decision to submit it for publication.

## Conflict of interest

RS-H is a co-founder of the CVAidMedical and MR is a consultant for the company. The remaining authors declare that the research was conducted in the absence of any commercial or financial relationships that could be construed as a potential conflict of interest.

## Publisher's note

All claims expressed in this article are solely those of the authors and do not necessarily represent those of their affiliated organizations, or those of the publisher, the editors and the reviewers. Any product that may be evaluated in this article, or claim that may be made by its manufacturer, is not guaranteed or endorsed by the publisher.

## References

[1] Goyal M Menon BK van Zwam WH Dippel DWJ Mitchell PJ Demchuk AM. Endovascular thrombectomy after large-vessel ischaemic stroke: a meta-analysis of individual patient data from five randomised trials. Lancet. (2016) 387:1723–31. 10.1016/S0140-6736(16)00163-X 26898852

[2] Albers GW Marks MP Kemp S Christensen S Tsai JP Ortega-Gutierrez S. Thrombectomy for stroke at 6 to 16 hours with selection by perfusion imaging. N Engl J Med. (2018) 378:708–18. 10.1056/NEJMoa1713973 29364767 PMC6590673

[3] Nogueira RG Jadhav AP Haussen DC Bonafe A Budzik RF Bhuva P. Thrombectomy 6 to 24 hours after stroke with a mismatch between deficit and infarct. N Engl J Med. (2018) 378:11–21. 10.1056/NEJMoa1706442 29129157

[4] Michel P. Prehospital scales for large vessel occlusion: closing in on a moving target. Stroke. (2017) 48:247–9. 10.1161/STROKEAHA.116.015511 28087805

[5] Ismail M Armoiry X Tau N Zhu F Sadeh-Gonik U Piotin M. Mothership versus drip and ship for thrombectomy in patients who had an acute stroke: a systematic review and meta-analysis. J Neurointerv Surg. (2019) 11:11–9. 10.1136/neurintsurg-2018-014249 30297541

[6] Saver JL Goyal M van der Lugt A Menon BK Majoie CBLM Dippel DW. Time to treatment with endovascular thrombectomy and outcomes from ischemic stroke: a meta-analysis. JAMA. (2016) 316:1279. 10.1001/jama.2016.13647 27673305

[7] Mohamad NF Hastrup S Rasmussen M Andersen MS Johnsen SP Andersen G. Bypassing primary stroke centre reduces delay and improves outcomes for patients with large vessel occlusion. Eur Stroke J. (2016) 1:85–92. 10.1177/2396987316647857 31008269 PMC6301230

[8] Powers WJ Rabinstein AA Ackerson T Adeoye OM Bambakidis NC Becker K. 2018 guidelines for the early management of patients with acute ischemic stroke: a guideline for healthcare professionals from the american heart association/american stroke association. Stroke. (2018) 49:e46–110. 10.1161/STR.0000000000000158 29367334

[9] Larsen K Jæger HS Hov MR Thorsen K Solyga V Lund CG. Streamlining acute stroke care by introducing national institutes of health stroke scale in the emergency medical services: a prospective cohort study. Stroke. (2022) 53:2050–7. 10.1161/STROKEAHA.121.036084 35291821 PMC9126266

[10] Smith EE Kent DM Bulsara KR Leung LY Lichtman JH Reeves MJ. Accuracy of prediction instruments for diagnosing large vessel occlusion in individuals with suspected stroke: a systematic review for the 2018 guidelines for the early management of patients with acute ischemic stroke. Stroke. (2018) 49:e111–22. 10.1161/STR.0000000000000160 29367333

[11] Takao H Sakai K Mitsumura H Komatsu T Yuki I Takeshita K. A smartphone application as a telemedicine tool for stroke care management. Neurol Med Chir. (2021) 61:260–7. 10.2176/nmc.oa.2020-0302 33716234 PMC8048116

[12] Martins SCO Weiss G Almeida AG Brondani R Carbonera LA de Souza AC. Validation of a smartphone application in the evaluation and treatment of acute stroke in a comprehensive stroke center. Stroke. (2020) 51:240–6. 10.1161/STROKEAHA.119.026727 31847753

[13] Shrout PE Fleiss JL. Intraclass correlations: uses in assessing rater reliability. Psychol Bull. (1979) 86:420–8. 10.1037/0033-2909.86.2.420 18839484

[14] Specogna AV Patten SB Turin TC Hill MD. The reliability and sensitivity of the national institutes of health stroke scale for spontaneous intracerebral hemorrhage in an uncontrolled setting. PLoS ONE. (2013) 8:e84702. 10.1371/journal.pone.0084702 24367691 PMC3868650

[15] Alasheev AM Andreev AY Gonysheva YV Lagutenko MN Lutskovich OY Mamonova AV. A comparison of remote and bedside assessment of the national institute of health stroke scale in acute stroke patients. Eur Neurol. (2017) 77:267–71. 10.1159/000469706 28391278

[16] Anderson ER Smith B Ido M Frankel M. Remote assessment of stroke using the iPhone 4. J Stroke Cerebrovasc Dis. (2013) 22:340–4. 10.1016/j.jstrokecerebrovasdis.2011.09.013 22018507

[17] Demaerschalk BM Vegunta S Vargas BB Wu Q Channer DD Hentz JG. Reliability of real-time video smartphone for assessing national institutes of health stroke scale scores in acute stroke patients. Stroke. (2012) 43:3271–7. 10.1161/STROKEAHA.112.669150 23160878

[18] Shafqat S Kvedar JC Guanci MM Chang Y Schwamm LH. Role for telemedicine in acute stroke. Stroke. (1999) 30:2141–5. 10.1161/01.STR.30.10.2141 10512919

[19] Goldstein LB Bertels C Davis JN. Interrater reliability of the nih stroke scale. Arch Neurol. (1989) 46:660–2. 10.1001/archneur.1989.00520420080026 2730378

[20] Brott T Adams HP Olinger CP Marler JR Barsan WG Biller J. Measurements of acute cerebral infarction: a clinical examination scale. Stroke. (1989) 20:864–70. 10.1161/01.STR.20.7.864 2749846

[21] Van Hooff RJ De Smedt A De Raedt S Moens M Mariën P Paquier P. Unassisted assessment of stroke severity using telemedicine. Stroke. (2013) 44:1249–55. 10.1161/STROKEAHA.111.680868 23444305

[22] Wang S Lee SB Pardue C Ramsingh D Waller J Gross H. Remote evaluation of acute ischemic stroke. Stroke. (2003) 34:e188–91. 10.1161/01.STR.0000091847.82140.9D 14500929

[23] Smith SNC Govindarajan P Padrick MM Lippman JM McMurry TL Resler BL. A low-cost, tablet-based option for prehospital neurologic assessment. Neurology. (2016) 87:19–26. 10.1212/WNL.0000000000002799 27281534 PMC4932237

[24] Wu TC Nguyen C Ankrom C Yang J Persse D Vahidy F. Prehospital utility of rapid stroke evaluation using in-ambulance telemedicine: a pilot feasibility study. Stroke. (2014) 45:2342–7. 10.1161/STROKEAHA.114.005193 24938842 PMC4116449

